# Pleomorphic Adenoma of the Larynx: A Case Report 

**Published:** 2016-01

**Authors:** Seyyed Jafar Motahari, Fereshteh Khavarinejad, Shahram Salimi, Milad Bahari

**Affiliations:** 1 *Department of Otolaryngology, Mazandaran University Of Medical Sciences, Sari, Iran.*; 2 *Ghaemshahr Health Center, Department of Research, Iran.*; 3 *Department of Pathology, Mazandaran University Of Medical Sciences, Ghaemshahr, Iran.*; 4 *Medical Student Research Center, Faculty of Medicine, Student Research Committee, Mazandaran University Of Medical Sciences, Sari, Iran.*

**Keywords:** Pleomorphic adenoma, Trans hyoid pharyngotomy, Ventricle of the larynx.

## Abstract

**Introduction:**

Pleomorphic adenomas are tumors mostly originating from salivary glands. These lesions in the larynx are very rare.

**Case Report:**

We report a rare case of pleomorphic adenoma that originated from the mucosal lining, just above the glottic area at the level of the laryngeal ventricle in a 55-year-old female patient. The tumor could not be palpated easily but was observed in the CT scan. We resected the large and firm tumor using trans hyoid pharyngotomy as the surgical approach.

**Conclusion:**

Pleomorphic adenoma in the ventricle of the larynx is an extremely rare lesion. Trans-hyoid pharyngotomy can have good results as the surgical approach in removing such lesions.

## Introduction

Most pleomorphic adenomas originate from the major salivary glands, especially the parotid gland. However, minor salivary glands may be the site of origin for some cases of this tumor. Such lesions in the larynx are very rare and reported cases in literature are less than 30. When the larynx is involved with this benign tumor, the most common site of origin will be the epiglottis (subglottis in some articles), but in our case, we describe a mixed tumor from the ventricle of the larynx that is not recorded in literature as the site of origin. Minor salivary glands may be the site of origin for pleormorphic adenoma in different areas such as the oral cavity, pharynx, retromulararea, nose, sinuses, parapharyngeal space, lacrimalgland, and even the breast (1-6).

The total number of reported cases of laryngeal pleomorphic adenoma in literature are 23. In Cummings’ textbook of otolaryngology, the total reported cases are 27. In Boo-Ali-Sina medical center in Sari, Iran, another case of epiglottic mixed tumor is reported. Pleomorphic adenoma can originate in any part of the larynx, but some of the authors report the epiglottis and some of them report the subglottis as the most common site of this benign tumor (7,8). However, upon review of various articles, pleomorphic adenoma, originated from the mucosal lining, just above the glottic area at the level of laryngeal ventricle. This was also seen in our patient and is very rare. The treatment of choice is surgical removal of the tumor and current approaches are pharyngotomy, laryngofissure and also use of laser in some cases .We chose the transhyoid pharyngotomy as the surgical approach and the results of surgery, functions of the larynx, and general condition of our patient was excellent after ten year follow up. Cellular pleomorphic adenoma has three differential diagnoses pathologically. Cellular pleomorphic adenoma exhibits considerable morphologic overlap with polymorphous low-grade adenocarcinoma and adenoid cystic carcinoma, especially in small biopsy specimens (9).

## Case Report

**Fig1:**
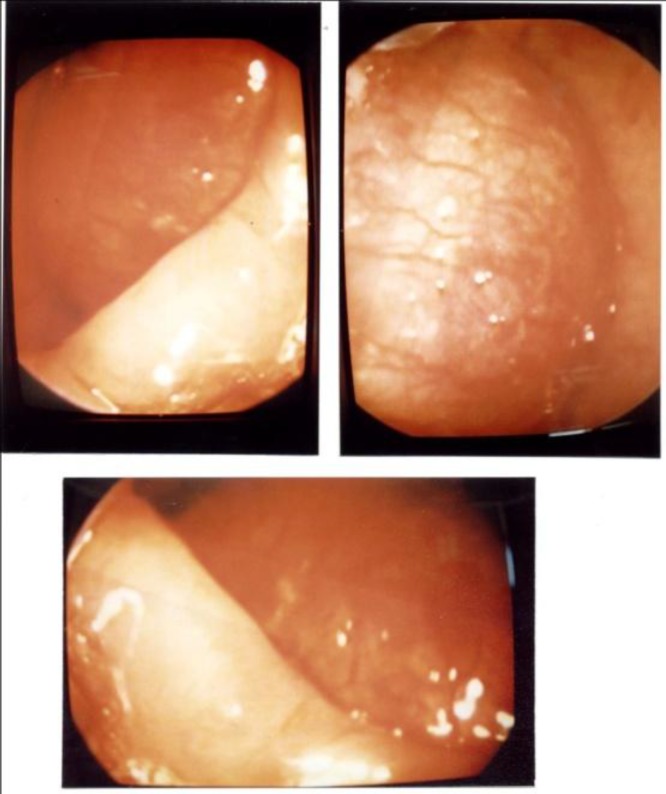
Gross view of the tumor

A 55-year-old female patient was brought to the emergency ward because of severe dyspnea and cyanosis and she went into the apnea state rapidly. During resuscitation and endotracheal intubation, an obstructing mass was seen in the laryngeal lumen and therefore she was intubated with great difficulty and then admitted to the ICU. She had dyspnea since 2 years, first in the exertional form and then even during rest. Six months before presenting with these symptoms she had a change of voice and dysphonia. When lying down on her right side, her dyspnea became more severe. She also complained of dysphagia but suffered no weight loss. She didn't smoke or drink alcohol and there was no history of infectious disease, or positive family history. During direct laryngoscopy and video laryngoscopy, a large and firm tumor was seen, filling about 90% of the intra laryngeal lumen. It was pink in color with a distinct and marked vascular pattern over its capsule (Fig.1).

**Fig 2:**
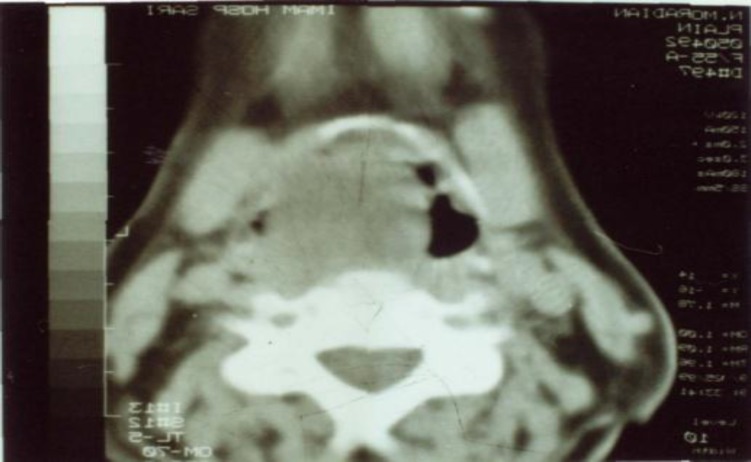
CT scan view of the tumor

The pedicle of this mobile tumor was toward the lower parts in the larynx and could not be palpated easily. In the CT scan, the tumoral lesion was seen from the ventricle and above the base of the epiglottis (Fig.2).

**Fig3:**
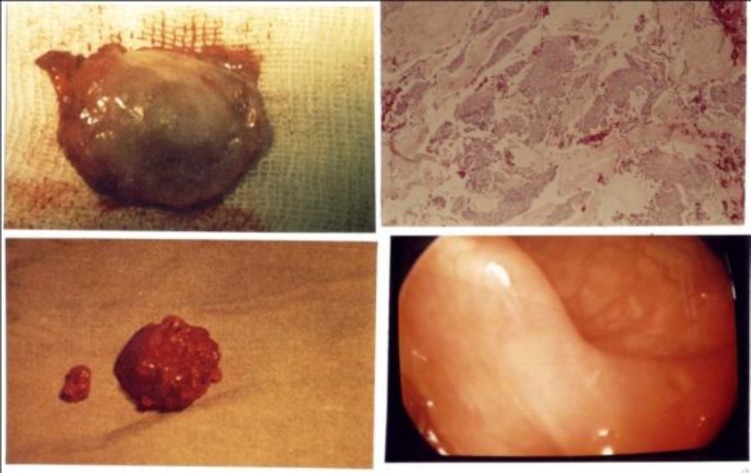
Pathologic view of the tumor

The excised tumor was an encapsulated tumor with a mucosal lining and its size was about 3.3 x 5.4 centimeters (Fig.3).

**Fig 4:**
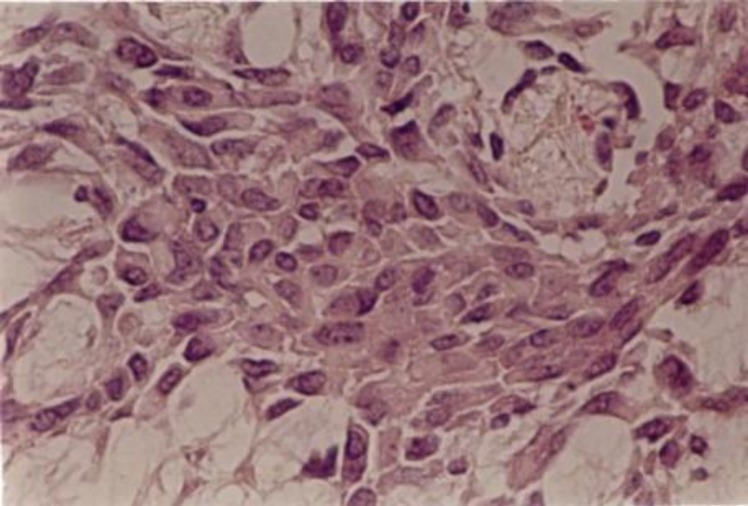
Microscopic view of the tumor

Upon microscopic description, groups of epithelial cells in a mixoid ground were reported (Fig. 4).

Although the diagnosis of pleomorphic adenoma is easily possible with light microscopy in most cases, some special immunohistochemical stainings are also helpful (10). Mitotic features are not common in the lesion. Pseudoepitheli- omatous hyperplasia in the mucosal lining of a mixed tumor in the oral cavity was reported in an article (11). Also there is a report of myoepithelial carcinoma within a tumor accompanied by a pathologic diagnosis of a pleomorphic adenoma in the buccal mucosa (12). Fortunately, there were no similar features in the pathologic description of the presented case.

## Discussion

Upon review of literature, 27 cases of pleomorphic adenoma of the larynx were reported up to 2011. Like any other benign laryngeal tumor, symptoms of laryngeal mixed tumors include dyspnea, dysphonia or hoarseness, and dysphagia (7,8). Respiratory distress at the beginning of the presentation may be exertional, but later, with increase in the size of the tumor, will present at rest and may be exacerbated in some positions. There is a change of voice in about half of patients (8). According to some reports and articles, one half of the laryngeal mixed tumors originate in the epiglottis, and the epiglottis is mentioned as the most common site of this tumor in the larynx. Because of non-specific symptoms in this disease, there is usually a relatively long time of delay for definitive diagnosis. Although for tracheal benign tumors symptoms occur after many years (10), the same tumors in the larynx with this exact time of delay are not recorded. The delay for our patient was about 2 years. She was being treated for exertional dyspnea and respiratory difficulties during sleep and even for asthma. Therefore, a complete head and neck examination including indirect or direct laryngoscopy with regard to the benign tumors in this organ is necessary for patients with respiratory problems. Histopathological examination is considered for diagnosis before surgical excision in all reports. There are some questions about the risk of recurrence and seeding of the pleomorphic adenoma in the larynx and this should be considered in performing incisional biopsy and or endoscopic excision of a small lesion. With more cases during the following years, these questions can be answered. In most reports, the treatment of choice is surgical excision.

Current approaches in use for the excision of laryngeal pleomorphic adenomas include lateral pharyngotomy, laryngofissure, and use of laser (7,13). Epiglottectomy through lateral pharyngotomy was reported in a case of an epiglottic mixed tumor (7). In our patient, the large tumor was filling the lumen. Therefore, the central portion of the hyoid bone was resected, trans-hyoid pharyngotomy was performed, the epiglottis was pulled out anteriorly with fine retractors, the lumen of the larynx was exposed, and the pedicle of the tumor was found with difficulty so the encapsulated tumor was resected in an en-block fashion. Despite the vascular nature over the capsule, there was no hemorrhage. After excision of the tumor, the wound was closed in related layers.

Cryotherapy was also used for treating the same lesions in the trachea. Possible complications in each of these approaches are recorded. Bilateral recurrent nerve paralysis was reported in a case treated with laser. In our patient, after transhyoid pharynogotomy a pharyngo cutaneous fistula was treated successfully with conservative procedures including aspirations and pressure dressings. Although complete surgical excision with a safe margin of normal tissue is the treatment of choice, the chance of recurrence cannot be ignored and the patient should be observed and followed in regular post op visits. In most cases, recurrence occurred 18 months after surgery (8). The main cause of recurrence is considered to be incomplete excision in the first surgery (8). An important point is the probability of malignancy in the lesion as the cause of recurrence. Complete surgical excisions of the tumor prevent the small risk of its malignant degeneration(10). Despite the fact that the laryngeal mixed tumor is a benign lesion, there are some reports of metastasis of this tumor to the cervical lymph nodes and other organs (8). Incomplete surgery of the primary lesion and other repeated excisions reported before metastases have been known to appear (14). Finally, the role of radiation therapy may be considered in rare cases of laryngeal mixed tumors. These cases include incomplete resection of the primary lesion or in cases with contraindications for surgery because of their general conditions (10). There is no report about the role of radiation therapy in recurrent lesions.

## Conclusion

In any case of hoarseness and/or shortness of breath, all parts of the larynx should be considered as the site of origin of a mass lesion. In benign tumors of the laryngeal ventricle, trans-hyoid pharyngotomy can be a good approach with acceptable results.
